# Supplementary material for: Observation of supersymmetry and its spontaneous breaking in a trapped ion quantum simulator
Source: Nat Commun. 2022 Jun 14;13:3412. doi: 10.1038/s41467-022-31058-0 (PMC9197856; doi:10.1038/s41467-022-31058-0)
Supplement: Supplementary file 1 — Supplementary Information [file 41467_2022_31058_MOESM1_ESM.pdf]

Supplementary Information for "Observation of Supersymmetry and its Spontaneous Breaking in a Trapped Ion Quantum Simulator"

M.-L. Cai,[1,2,*] Y.-K. Wu,[1,*] Q.-X. Mei,[1] W.-D. Zhao,[1] Y. Jiang,[1] L. Yao,[1,2] L. He,[1] Z.-C. Zhou,[1,3] and L.-M. Duan[1,†]

[1] *Center for Quantum Information, Institute for Interdisciplinary Information Sciences, Tsinghua University, Beijing, 100084, PR China*

[2] *HYQ Co., Ltd., Beijing, 100176, PR China*

[3] *Beijing Academy of Quantum Information Sciences, Beijing 100193, PR China*

SUPPLEMENTARY NOTE 1: SUPERSYMMETRY IN QUANTUM RABI MODEL

A quantum mechanical system is supersymmetric if we can define some Hermitian supercharges $Q_1, Q_2, \cdots, Q_N$ such that $\{Q_i, Q_j\} = 2H\delta_{ij}$, where H is the Hamiltonian of the system [1]. By definition, all the supercharges commute with $H = Q_i^2$, so that Q_i's are conserved quantities.

The simplest case is the $N = 2$ SUSY QM with two supercharges. Here we can define a Witten parity operator K satisfying $K^2 = I$ and $\{K, Q_i\} = 0$ $(i = 1, 2)$. It can be shown that for the $N = 2$ SUSY QM we can choose $Q_2 = \pm iKQ_1$, so that we only need to consider one supercharge [1]. From the above definitions, we can see that $[H, K] = 0$, hence K is also conserved. We can thus split the whole Hilbert space into $\mathcal{H} = \mathcal{H}_+ \oplus \mathcal{H}_-$ where $\mathcal{H}_\pm$ is the eigenspace of K with eigenvalue ± 1 which corresponds to the bosonic and the fermionic states, respectively.

Now we consider the QRM model [Eq. (1) of the main text], which is supersymmetric at two sets of parameters [2]: $g = 0$ and $\omega_s = \omega$; or $\omega_s = 0$.

A. $g = 0$ and $\omega_s = \omega$

In this case, the Hamiltonian $H = \omega\sigma_z/2 + \omega(a^\dagger a + 1/2)$ is just a spin and a bosonic mode without any interaction. It is easy to check that $Q = \sqrt{\omega}(a\sigma_+ + a^\dagger\sigma_-)$ and $K = \sigma_z$ satisfy all the above definitions. Here we can see that the spin-up and the spin-down states with different phonon numbers give the whole spectrum of the bosonic and the fermionic states, and the mapping from spin-up/spin-down to bosonic/fermionic states is arbitrary: We can simply define $K \to -K$ to reverse the Witten parity operator.

Let us choose $|\downarrow\rangle|n\rangle$ as the bosonic states and $|\uparrow\rangle|n\rangle$ as the fermionic states. Here we have a unique ground state $|\downarrow\rangle|0\rangle$ with energy $E_0 = 0$, and the higher levels $|\downarrow\rangle|n+1\rangle$ and $|\uparrow\rangle|n\rangle$ are degenerate with energy $E_{n+1} = (n+1)\hbar\omega$ $(n = 0, 1, \cdots)$. The supercharge Q transforms the degenerate bosonic and fermionic states into each other with a nonzero normalization factor, and it annihilates the unique ground state.

B. $\omega_s = 0$

In this case the Hamiltonian is given by

$$H = \omega\left(a^\dagger a + \frac{1}{2}\right) + g\sigma_x(a + a^\dagger) + \frac{g^2}{\omega}. \tag{1}$$

To demonstrate the SUSY structure more clearly, we follow Ref. [2] to perform a unitary transform

$$U_g = \frac{1}{\sqrt{2}}\begin{pmatrix} V_- & -V_+ \\ V_- & V_+ \end{pmatrix}, \tag{2}$$

where $V_\pm = \exp[\pm g/\omega(a^\dagger - a)] = D(\pm g/\omega)$ is the displacement operator of $\pm g/\omega$. It is straightforward to verify that

$$U_g^\dagger H U_g = \omega I \otimes \left(a^\dagger a + \frac{1}{2}\right). \tag{3}$$

* These authors contribute equally to this work

† lmduan@mail.tsinghua.edu.cn

A supercharge in this transformed frame can be given by

$$U_g^\dagger Q U_g = \sigma_x \otimes \sqrt{\omega(a^\dagger a + 1/2)}, \tag{4}$$

with the Witten parity operator $U_g^\dagger K U_g = \sigma_z \otimes I$. Moving back to the original frame, we get

$$\begin{aligned} Q =& U_g \sigma_x \otimes \sqrt{\omega(a^\dagger a + 1/2)} U_g^\dagger \\ =& -\frac{\sigma_z}{2} \otimes \left[V_+ \sqrt{\omega(a^\dagger a + 1/2)} V_+ + V_- \sqrt{\omega(a^\dagger a + 1/2)} V_- \right] \\ & - i\frac{\sigma_y}{2} \otimes \left[V_+ \sqrt{\omega(a^\dagger a + 1/2)} V_+ - V_- \sqrt{\omega(a^\dagger a + 1/2)} V_- \right], \end{aligned} \tag{5}$$

and $K = \sigma_x$. In the main text, we have taken out the factor $\sqrt{\omega}$ from the definition of Q to make it dimensionless. Then we have $H = \omega Q^2$.

The ground state in the transformed frame has energy $E_0 = \omega/2$ when the phonon number is zero. If we further require the states to be the eigenstates of Q, we see that, in the transformed frame, the two ground states can be chosen as $|\pm\rangle|0\rangle$, with eigenvalues of $\pm 1/\sqrt{2}$ for the supercharge. Now if we move back to the original frame, the two ground states can be expressed as $U_g|\pm\rangle|0\rangle = (|+\rangle|-g/\omega\rangle \mp |-\rangle|g/\omega\rangle)/\sqrt{2}$.

SUPPLEMENTARY NOTE 2: COMPLETE EXPERIMENTAL DATA FOR PARTIAL SPIN-PHONON STATE TOMOGRAPHY

In Supplementary Figure 1, Supplementary Figure 2, Supplementary Figure 3 and Supplementary Figure 4 we present the complete experimental data for measuring the joint spin-phonon state by projecting $|\psi_\pm\rangle$ onto $\sigma_z = \pm 1$ or $\sigma_y = \pm 1$, as well as the theoretical results for the best fitted joint density matrices.

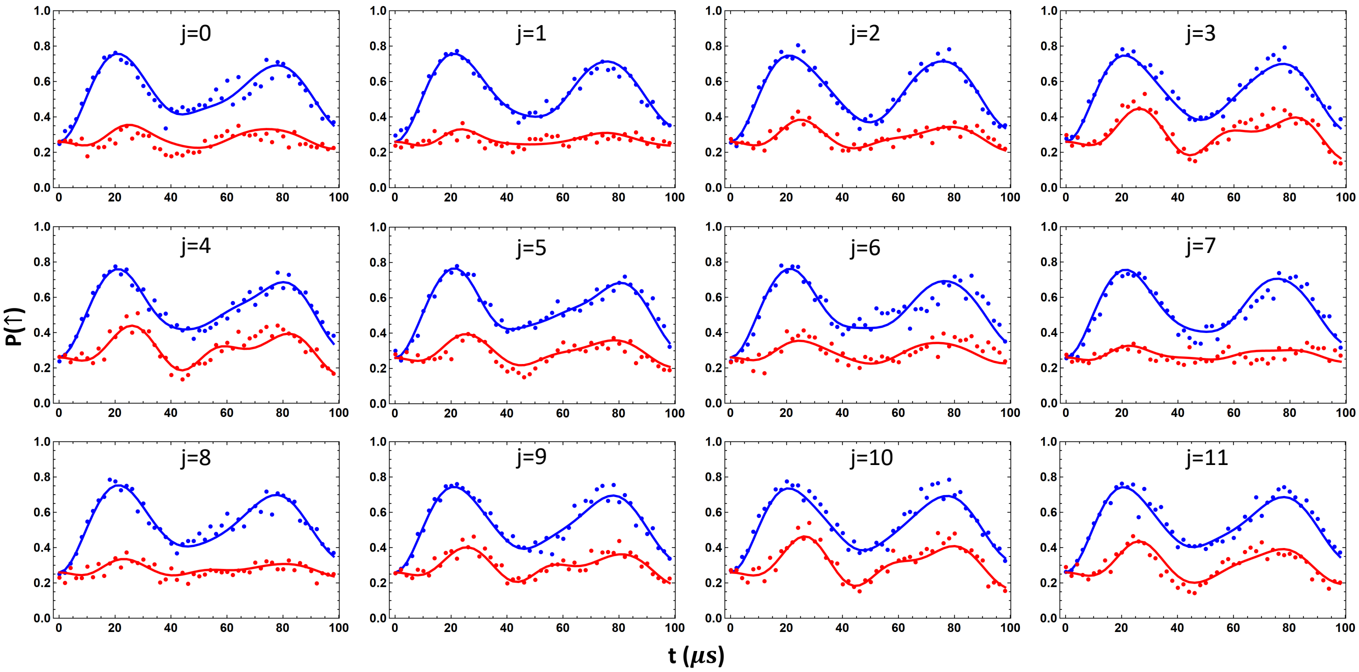

Supplementary Figure 1. Experimental data (dots) and the theoretical prediction based on the best-fitted density matrix (solid curves) when driving the blue or red sidebands for the $|\psi_-\rangle$ state projected to $\sigma_z = \pm 1$. We choose $\omega = 2\pi \times 10\,\text{kHz}$ and $g_m = 2\pi \times 5.43\,\text{kHz}$. The displacement operators $D(\beta_j)$ are characterized by $\beta_j = i\beta e^{2\pi i j/N}$ where $\beta = 0.687$, $N = 12$ and $j = 0, 1, \cdots, N-1$.

In Supplementary Figure 5 and Supplementary Figure 6 we present the experimental data used to calibrate a phonon state rotation angle between the measurement of $|\psi_+\rangle$ and $|\psi_-\rangle$ as described in Methods.

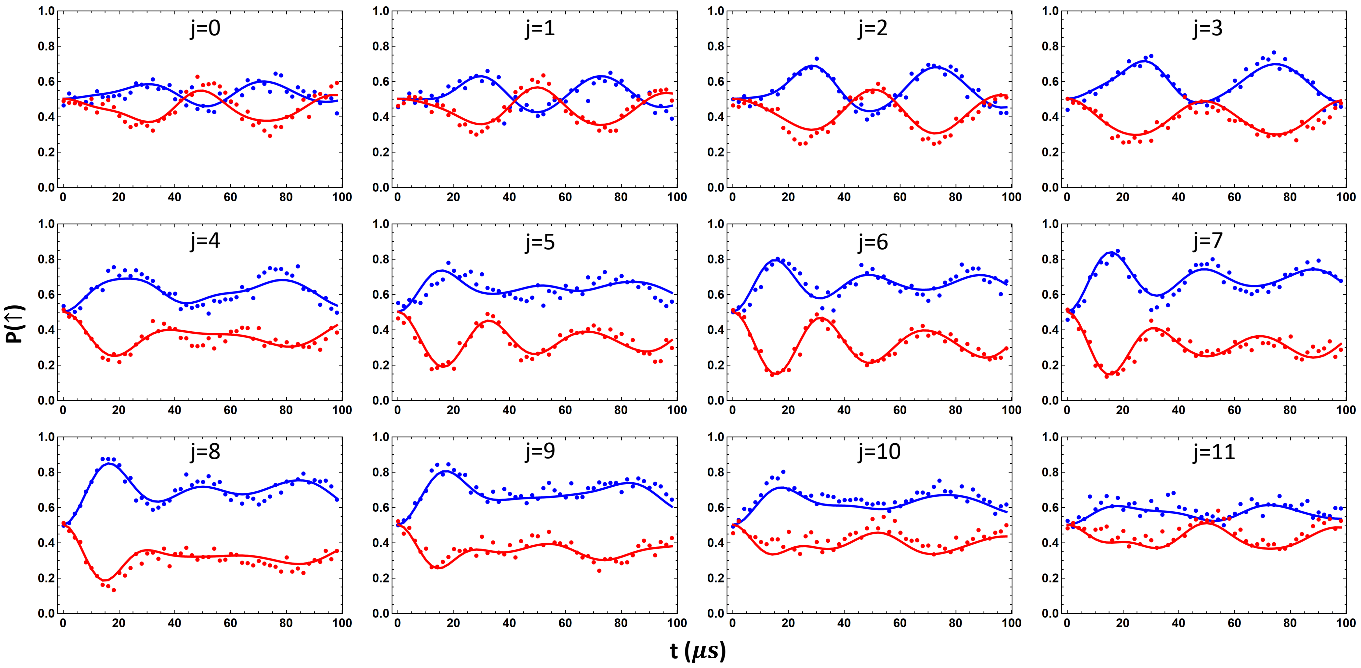

Supplementary Figure 2. Experimental data (dots) and the theoretical prediction based on the best-fitted density matrix (solid curves) when driving the blue or red sidebands for the $|\psi_-\rangle$ state projected to $\sigma_y = \pm 1$. We choose $\omega = 2\pi \times 10\,\mathrm{kHz}$ and $g_m = 2\pi \times 5.43\,\mathrm{kHz}$. The displacement operators $D(\beta_j)$ are characterized by $\beta_j = i\beta e^{2\pi i j/N}$ where $\beta = 0.687$, $N = 12$ and $j = 0, 1, \cdots, N-1$.

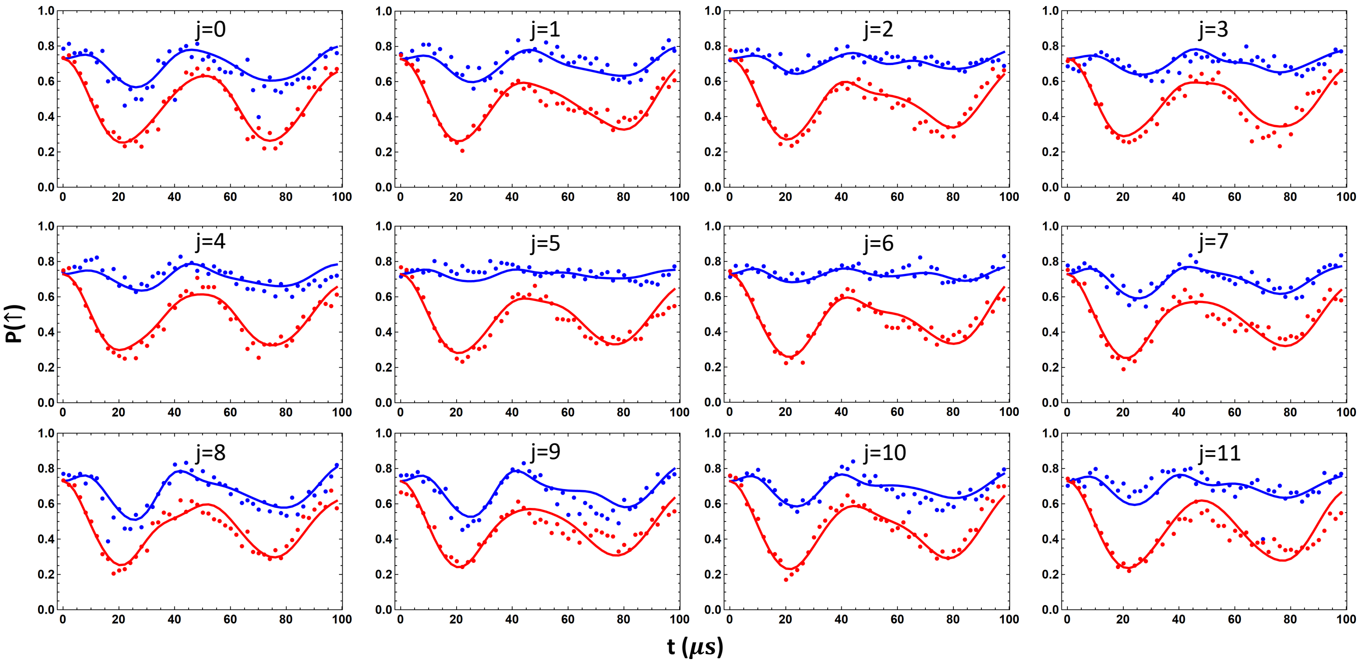

Supplementary Figure 3. Experimental data (dots) and the theoretical prediction based on the best-fitted density matrix (solid curves) when driving the blue or red sidebands for the $|\psi_+\rangle$ state projected to $\sigma_z = \pm 1$. We choose $\omega = 2\pi \times 10\,\mathrm{kHz}$ and $g_m = 2\pi \times 5.43\,\mathrm{kHz}$. The displacement operators $D(\beta_j)$ are characterized by $\beta_j = i\beta e^{2\pi i j/N}$ where $\beta = 0.687$, $N = 12$ and $j = 0, 1, \cdots, N-1$.

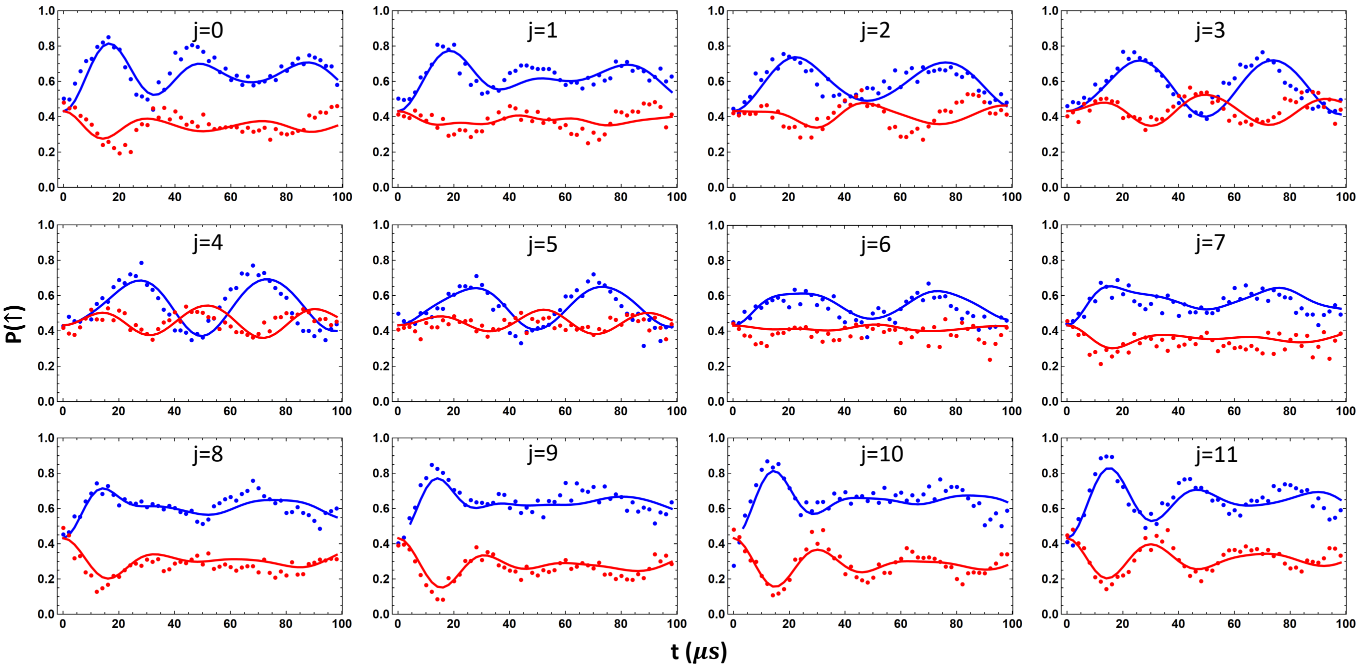

Supplementary Figure 4. Experimental data (dots) and the theoretical prediction based on the best-fitted density matrix (solid curves) when driving the blue or red sidebands for the $|\psi_+\rangle$ state projected to $\sigma_y = \pm 1$. We choose $\omega = 2\pi \times 10\,\text{kHz}$ and $g_m = 2\pi \times 5.43\,\text{kHz}$. The displacement operators $D(\beta_j)$ are characterized by $\beta_j = i\beta e^{2\pi i j/N}$ where $\beta = 0.687$, $N = 12$ and $j = 0, 1, \cdots, N-1$.

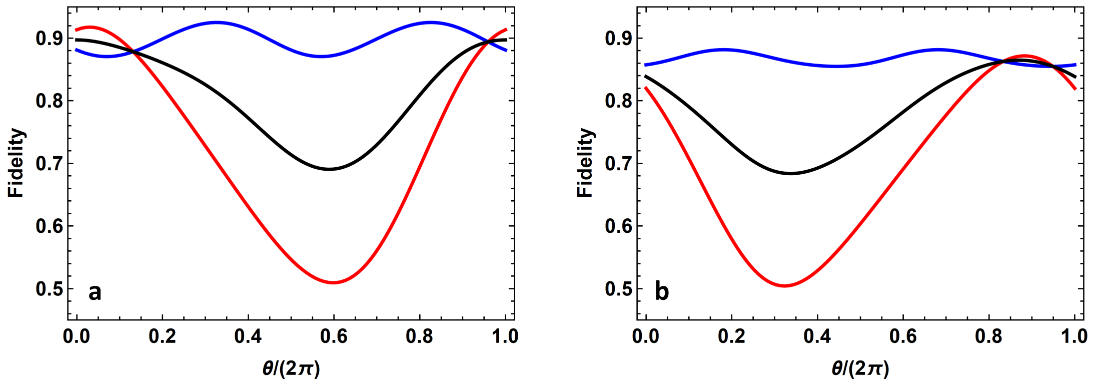

Supplementary Figure 5. Fidelity for the spin-phonon state projected to $\sigma_z = \pm 1$ (blue), $\sigma_y = \pm 1$ (red) and their average (black) for **a** $|\psi_-\rangle$ and **b** $|\psi_+\rangle$.

[1] Combescure, M., Gieres, F. & Kibler, M. Are N= 1 and N= 2 supersymmetric quantum mechanics equivalent? *Journal of Physics A: Mathematical and General* **37**, 10385–10396 (2004).

[2] Hirokawa, M. The Rabi model gives off a flavor of spontaneous SUSY breaking. *Quantum Studies: Mathematics and Foundations* **2**, 379–388 (2015).

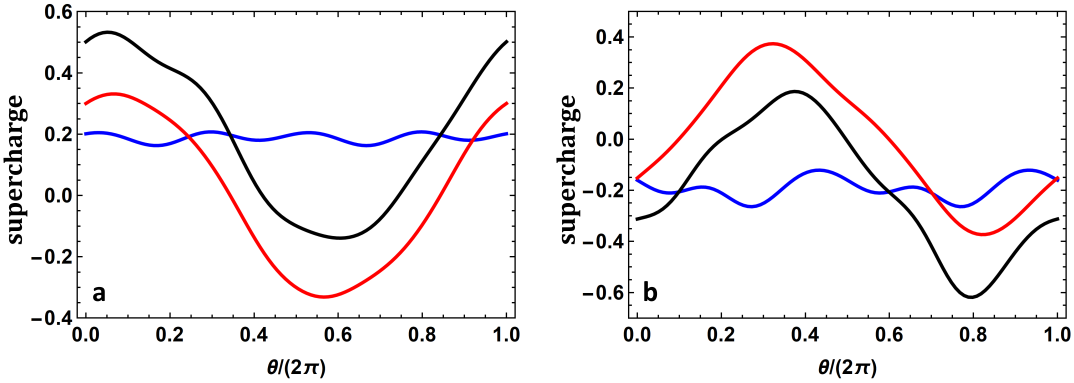

Supplementary Figure 6. Expectation values for $\sigma_z \otimes A$ (blue), $\sigma_y \otimes B$ (red) and the total supercharge (black) for **a** $|\psi_-\rangle$ and **b** $|\psi_+\rangle$.
